# Effects of levetiracetam and oxcarbazepine monotherapy on intellectual and cognitive development in children with benign epilepsy with centrotemporal spikes

**DOI:** 10.1007/s13760-021-01613-5

**Published:** 2021-02-15

**Authors:** Gui-hai Suo, Yu-qin Zheng, You-jia Wu, Ji-hong Tang

**Affiliations:** 1 Department of Pediatrics, Children’s Hospital of Soochow University & Affiliated Hospital of Nantong University, No. 92 Zhongnan Street, Suzhou Industrial Park, 215025 Jiangsu China; 2 Department of Pediatrics, Affiliated Hospital of Nantong University, No.20 Xi Si Road, Nantong, 226001 Jiangsu China; 3 Department of Pediatrics, Children’s Hospital of Soochow University, No. 92 Zhongnan Street, Suzhou Industrial Park 215025, Jiangsu, China

**Keywords:** Levetiracetam (LEV), Oxcarbazepine (OXC), Benign epilepsy with centrotemporal spikes (BECTS), Efficacy, Intelligence, Cognitive development

## Abstract

Levetiracetam (LEV) and oxcarbazepine (OXC) are commonly used in the treatment of epilepsy, but their efficacy and safety have seldom been compared for the treatment of children with benign epilepsy with centrotemporal spikes (BECTS). We thus assessed the efficacy of LEV and OXC monotherapy in the treatment of children with BECTS, and the effect of this treatment on children’s intelligence and cognitive development. This was a randomized, single-center trial. Children with BECTS were randomized (1:1) into LEV and OXC groups, and were assessed at 1, 3 and 6 months after treatment. The primary outcomes were the frequency of seizures and changes in intelligence and cognitive function. Secondary outcomes were electroencephalogram (EEG) results and safety. Seventy children were enrolled and randomized to the LEV group or the OXC group, and 32 of the 35 children in each group completed the study. After 6 months, the effective treatment rate of the OXC group was significantly higher than that of the LEV group (78.12 vs. 53.12%, *p* = 0.035). However, no significant inter-group difference was observed in EEG improvement (*p* = 0.211). In terms of intelligence and cognitive development, children in the OXC group exhibited significantly improved choice reaction time, mental rotation, and Wisconsin Card Sorting Test results (all *p* < 0.05). Both LEV and OXC were well tolerated, with 18.75 and 21.88% of children reporting mild adverse events (*p* = 0.756). OXC monotherapy was more effective than LEV for children with BECTS. In addition, children with OXC monotherapy had higher improvements in children’s intelligence and cognitive function than those with LEV monotherapy.

## Introduction

Epilepsy is a chronic transient brain dysfunction syndrome caused by the abnormal electrical discharge from neurons in the brain that may affect children’s physical, intellectual and mental health [1]. Benign epilepsy with centrotemporal spikes (BECTS), also known as benign Rolandic epilepsy, is considered the most common benign epileptic syndrome in childhood [2], as it accounts for 15–24% of all cases of childhood epilepsy [3]. BECTS is an idiopathic focal epilepsy syndrome that has a good prognosis, and generally does not affect growth and development [4]. However, recent research has shown that children with BECTS have more learning and behavioral problems than their unaffected peers, and that epileptogenic discharge can damage the brain, and thus possibly affect intellectual development [5]. It is therefore recommended that children diagnosed with epilepsy should receive regular antiepileptic treatment as soon as possible.

Antiepileptic medication is essential for patients who have frequent attacks (i.e., ≥ 2 attacks per year), partial daytime attacks and secondary systemic attacks, and have a young age at onset. The choice of antiepileptic drugs (AEDs) for such patients is also more conservative [6]. Levetiracetam (LEV), as a typical second-generation AED for BECTS, is a derivative of pyrrolidone that selectively combines with synaptic vesicle protein 2A to promote vesicle aggregation and subsequent vesicle discharge from cells. This regulates neurotransmitter release and inhibits abnormal electrical discharge from neurons, thereby resulting in antiepileptic effects [7]. LEV has a broad antiepileptic spectrum and its indications and adaptive population are gradually expanding [8]. Oxcarbazepine (OXC) is also a second-generation AED, and is a derivative of carbamazepine. The mechanism of antiepileptic activity of OXC is similar to that of carbamazepine, although OXC is a prodrug, and its antiepileptic effects are exerted by its primary metabolite, a 10-mono-hydroxyl derivative, which is generated by the action of an aromatic ketone degrading enzyme on OXC [9]. Due to its low induction by liver enzymes, high bioavailability and low protein-binding rate [10], OXC has been suggested to have similar efficacy to carbamazepine, but superior tolerability and safety [11].

Currently, there were few studies found with few antiepileptic drugs compared for treatment of BECTS in Children [12]. Only an open-label, parallel group trial conducted by Coppola G et al. 12 has evaluated the efficacy and tolerability of LEV or OXC as monotherapy in 21 children with newly diagnosed BECTS. In addition, they did not assess their effects on the development of children’s intelligence and cognitive ability. Accordingly, there is still lack of evidence on comparison between LEV and OXC for children with BCECTS. Thus, a more comprehensive study is required. Therefore, in this study, we examined the efficacy of LEV and OXC monotherapy for the treatment of children with BCECTS, and the effect of these treatments on intelligence and cognitive development using a randomized controlled trial.

## Methods

### Study population

Children diagnosed with BECTS in the outpatient department of the Affiliated Hospital of Nantong University from October 2018 to February 2020 were selected as the research subjects. The inclusion criteria were (1) a diagnosis in line with the BECTS diagnostic criteria in the new (2017) International Anti-Epileptic Alliance classification of seizures and epilepsy [13]; (2) electroencephalogram (EEG) features that showed that a seizure during an epileptic attack was partial, or was generalized to the whole body, and that the background rhythm was normal; (3) at least two convulsions before enrollment; (4) no abnormality in head magnetic resonance imaging (MRI) or computed tomography examination; and (5) normal liver and kidney function prior to the commencement of medication. The exclusion criteria were (1) the presence of encephalitis, brain injury, cerebral hemorrhage, and other organic diseases of the nervous system; (2) functional insufficiency of the liver, lung, kidney or other important organs; (3) the emergence of mental retardation; (4) the presence of cranial space-occupying lesions; (5) poor medication compliance; and (6) the presence of relevant drug contraindications. Head scans were performed on all children using MRI to differentiate them from other related seizures.

The study was approved by the ethics committee of the Affiliated Hospital of Nantong University, and the parents or guardians of the eligible children signed informed consent forms.

### Grouping and treatment

This was a randomized clinical trial. The eligible children were randomized (1:1) via the random number table to the LEV group or the OXC group. Follow-up results were recorded at 1, 3 and 6 months after the commencement of treatment. During follow-up assessments, the frequency and form of seizures, EEG changes, cognitive changes, adverse drug events and drug tolerance data were recorded.

The LEV group was given Keppra (a brand of LEV manufactured by UCB Pharmaceuticals), in the form of tablets containing 250 mg of LEV. The initial dose was set at 10 mg/kg/day. Thereafter, it is advised that this dose is increased once every 7 days, and is maintained at 20–60 mg/kg/day. The OXC group was given OXC (manufactured by Novartis Pharma Schweiz AG), in the form of tablets containing 150 mg of OXC. The initial dose was set at 8–10 mg/kg/day, orally administered twice a day at an interval of 12 h. Thereafter, it is advised to increase the dose to 5–10 mg/kg/day every 5–7 days, depending on the situation, and maintain the dosage at 20–46 mg/kg/day. All children started treatment at a low dose and returned to the clinic for assessment once a week at the beginning of treatment. During the treatment, clinical reactions in each child were closely observed, and the dosage of drug was appropriately adjusted according to the weight of the child and his/her seizure status. If a child exhibited obvious adverse events, the treatment was adjusted. Both groups were followed up for 6 months.

Before the start of the study, the investigators explained the significance of the study to the subjects and explained the importance of taking medication on time to ensure the efficacy. In addition, the investigators called weekly to inquire about medication intake of each subject. Moreover, all the drug packages sent to the patients were returned to the researchers after the trial was over. According to the packages of used and remaining drugs, we could calculate the medication compliance of research subjects.

### Outcome measures

The monthly frequency of epileptic seizures in the 2 months before treatment was used as the standard, and the monthly frequency of epileptic seizures at 3 and 6 months after treatment was compared between the two groups. A curative outcome was a ≥ 95% decrease in the monthly seizure frequency after treatment. An effective treatment outcome was a 50–74% reduction in the frequency of monthly seizures, while an ineffective treatment outcome was a < 50% decrease or an increase in monthly seizure frequency. The results showed that the monthly frequency of seizures was reduced by 75 to < 95%. The total effective rate of seizure treatment was calculated as [(cured cases + apparently effective cases + effective cases)/total number of cases] × 100.

The amount of interictal epileptiform activity was recorded by EEG before and after treatment in the baseline stationary state, when a child was supine, awake and quiet, and was classified as (1) normal (the epileptic discharge had disappeared completely after treatment); (2) significantly improved: (the epileptic discharge was reduced by 50% compared with that before treatment); (3) improved (the epileptic discharge was reduced by less than 50% compared with that before treatment; (4) not improved (the EEG results showed no improvement compared to those before treatment); or (5) worsened (the EEG results showed that epileptic discharge was increased compared to that before treatment). The total response rate of EEG treatment was calculated as [(normal + significant improvement + improvement)/total number of cases] × 100.

The criteria used for the evaluation of cognitive function efficacy comprised eight indicators, and both groups were assessed according to these criteria before and after treatment. The neuropsychological assessment battery included eight cognitive tests that assessed multiple cognitive abilities: processing speed, spatial skills, calculation, language ability (including semantic comprehension and phonological processing ability), intelligence, visual attention and executive function.

All tests were programmed using web-based applications in the Online Experimental Psychological System (www.dweipsy.com/lattice) [14]. In the examination room, patients were given a series of tests over two 45-min sessions. For each test, the interviewer had to give the instructions and then practice. Participants’ responses were automatically recorded and sent via the Internet to a server. The tests were conducted by trained interviewers. For processing speed, a white dot appeared to either the left or the right of a fixed cross on a black screen. Participants were asked to press Q if the dot appeared on the left, or P if the dot appeared on the right. A total of 30 trials were conducted sequentially and the time used was summed up to calculate the choice reaction time. For spatial skills, participants were asked to select which 3D image to match the top image from the bottom of the screen by pressing the Q or P key; Matching images can only be identified by mental rotation. Simple subtraction was conducted to assess calculation ability with 92 simple subtraction problems. Participants were asked to press the Q key to select the answer on the left and the P key to select the answer on the right. For each test of word semantics, a sentence was displayed in the middle of the computer screen with one word missing. Participants were asked to choose one of the two candidate words by pressing either the Q key or the P key. Raven’s Progressive Matrices test was used to evaluate figure reasoning ability. Participants must determine that the missing parts of the character will be completed. Two candidate answers are displayed side by side below each question. If the missing part is on the left, instruct the participant to press Q; If on the right, the P key is indicated. For visual tracing, several curves in a square intersect each other, starting at the left side of the square and ending at the right. Participants were asked to follow the target line from start to finish with only their eyes, and then mark the correct end. WCST is one of the most widely used performing functional tests. The task consists of a stimulus card and a response card; According to one of three principles (i.e., color, form, or quantity), the participant was instructed to flip the card so that the response card matched one of the stimulus cards.

### Statistical analysis

Inter-group comparisons were performed for age, frequency of attack, and intelligence test score data, such as means and standard deviations (SDs), using independent sample *t* tests. Intra-group comparisons were performed using paired *t* tests, and enumeration data, such as sex, seizure type, follow-up epileptic seizure data and EEG results, were expressed with respect to the total number of cases (percent) [*n* (%)]. Comparisons were made using the chi-square test or chi-square correction formula, or Fisher’s exact test. Statistical Package for Social Sciences (SPSS) 20.0 statistical software was used for data analysis, with *p* < 0.05 regarded as an indicator of a statistically significant difference.

## Results

### Patients

**Fig. 1:**
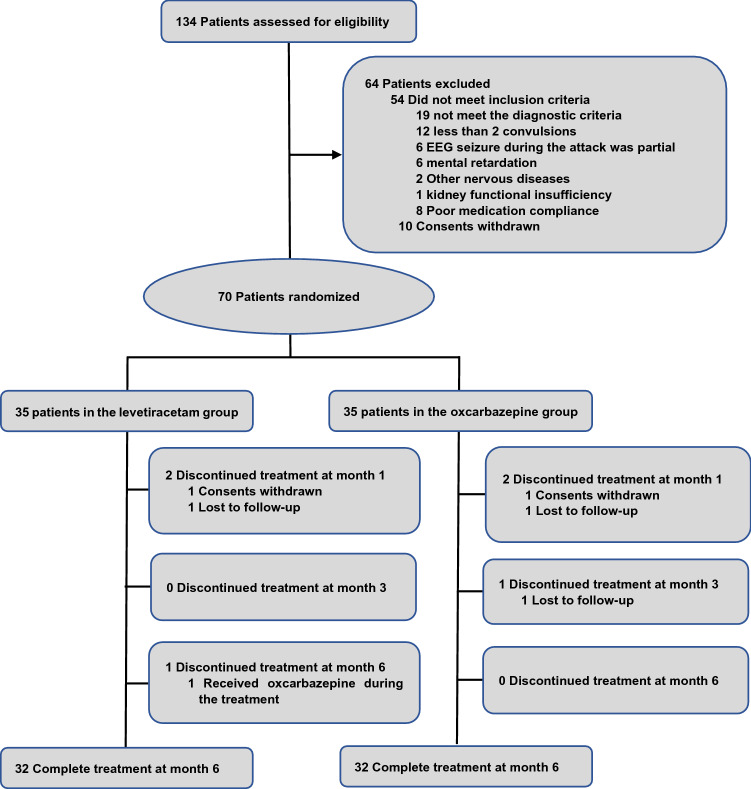
Flow chart of patient enrollment

Seventy children were randomized to the LEV (*N* = 35) group or the OXC (*N* = 35) group, and 32 children in each group were ultimately followed (Fig. 1).

The LEV group comprised 21 boys and 11 girls, who had an average age of 8.47 ± 2.13 years and an average age at onset of 6.98 ± 1.82 years. The OXC group comprised 19 boys and 13 girls, who had an average age of 8.62 ± 2.21 years and an average age at onset of 7.13 ± 1.75 years. There was no significant difference in sex, age, intelligence quotient, incidence frequency and type, EEG status, and family history between the two groups before formal enrollment (*p* > 0.05) (Table 1).

**Table 1:** Comparison of general conditions between two groups

	Levetiracetam (*n* = 32)	Oxcarbazepine (*n* = 32)	*p*
Age at survey, years	8.47 ± 2.13	8.62 ± 2.21	0.783
Age at onset, years	6.98 ± 1.82	7.13 ± 1.75	0.738
Gender, male%	21 (65.63)	19 (59.38)	0.606
Education, years	3.35 ± 0.61	3.43 ± 0.54	0.581
BMI, kg/m^2^	18.28 ± 2.25	18.70 ± 2.42	0.475
Seizures at baseline, times	2.74 ± 1.17	2.59 ± 1.05	0.591
Duration of epilepsy at baseline, months	7.09 ± 2.96	6.85 ± 2.57	
Seizure-type before enrollment			0.902
SP	8 (25.00)	10 (31.25)	
CP	5 (15.63)	4 (12.50)	
GTC	13 (40.62)	11 (34.38)	
P + (S)G	6 (18.75)	7 (21.87)	
Dosage, mg/kg/day	24.73 ± 8.49	27.04 ± 9.13	0.299
Family history of epilepsy, *n* (%)	4 (12.50)	7 (21.88)	0.320
History of febrile seizures, *n* (%)	3 (9.38)	1 (3.13)	0.606
Birth asphyxia, *n* (%)	3 (9.38)	4 (12.50)	1.000
EEG characteristics, *n* (%)			0.867
Focal specific discharges CT only	16 (50.00)	14 (43.75)	
Focal specific discharges CT-CFT	12 (37.50)	13 (40.63)	
Focal specific discharges CT-CPT	4 (12.50)	5 (15.62)	

*BMI* body max index, *CT* centrotemporal, *CPT* centroparietotemporal, *CFT* centrofrontotemporal, *SP* simple partial seizure, *CP* complex partial seizure, *GTC* generalized tonic–clonic seizure, *P + (S)G* complex partial seizure secondary with generalized tonic–clonic seizure

### Efficacy

At the 3-month follow-up, the effective treatment rate was 50% in the OXC group and 37.5% in the LEV group, but these clinical differences were not statistically significant (*p* = 0.313). After 6 months of follow-up, the effective treatment rate in the OXC group was 78.12%, while that in the LEV group was 53.12%, and these clinical differences were statistically significant (*p* = 0.035) (Table 2).

**Table 2:** Seizure and EEG outcome at the 3 and 6-month follow-up

	Levetiracetam (*n* = 32)	Oxcarbazepine (*n* = 32)	*p*
3 months			
Seizure outcome			0.313
Seizure	20 (62.50)	16 (50.00)	
Freedom	12 (37.50)	16 (50.00)	
EEG outcomes			0.434
Normal	10 (31.25)	13 (40.63)	
Abnormal	22 (68.75)	19 (59.37)	
6 months			
Seizure outcome			0.035
Seizure	15 (46.88)	7 (21.88)	
Freedom	17 (53.12)	25 (78.12)	
EEG outcomes			0.211
Normal	14 (43.75)	19 (59.38)	
Abnormal	18 (56.25)	13 (40.62)	

*EEG* Electroencephalography

### EEG

After 3 months of follow-up, the effective rate of EEG improvement was 40.63% in the OXC group and 31.25% in the LEV group. After 6 months of follow-up, the effective rate of EEG improvement was 59.38% in the OXC group and 43.75% in the LEV group. There was no significant difference between these rates of EEG improvement (*p* > 0.05) (Table 2).

### Intellectual and cognitive development

We used eight indicators to compare the children’s intelligence and cognitive function development (Table 3). Compared with pre-intervention, word semantics, Raven’s Progressive Matrices, visual tracing, and Wisconsin Card Sorting Test were significant improved after intervention for patients in both LEV and OXC group, and significant improvement were further observed for choice reaction time and mental rotation for patients in OXC group (all *p* < 0.05). In terms of processing speed (choice reaction time) (*p* = 0.038), spatial skills (mental rotation) (*p* = 0.034) and executive function (WCST) (*p* = 0.046), the OXC group improved more than the LEV group.

**Table 3:** Intelligence comparison between the two groups before and after the intervention

Test	Levetiracetam (*n* = 32)					Oxcarbazepine (*n* = 32)					*t2*	*p2*
	Pre-intervention	Post-intervention	Diff	*t1*	*p1*	Pre-intervention	Post-intervention	Diff	*t1*	*p1*		
Choice reaction time	512.6 ± 74.5	493.7 ± 70.56	18.9 ± 70.02	1.042	0.301	521.8 ± 79.8	465.5 ± 71.5	56.3 ± 75.05	2.972	**0.004**	2.034	**0.038**
Mental rotation	11.8 ± 4.5	13.5 ± 4.2	− 1.7 ± 4.01	1.562	0.123	12.6 ± 4.0	16.5 ± 4.5	− 3.9 ± 4.12	3.664	**0.001**	2.165	**0.034**
Simple subtraction	33.7 ± 5.6	36.0 ± 5.1	− 2.3 ± 5.04	1.718	0.091	33.2 ± 5.4	35.3 ± 5.0	− 2.1 ± 5.11	1.614	0.112	0.946	0.348
Word semantics	17.5 ± 3.8	22.0 ± 4.1	− 4.5 ± 3.92	4.554	** < 0.001**	18.3 ± 3.3	21.2 ± 3.7	− 2.9 ± 3.42	3.309	**0.002**	1.740	0.087
Raven’s progressive matrices	12.0 ± 4.8	15.7 ± 5.1	− 3.7 ± 4.88	2.989	**0.004**	11.6 ± 4.1	14.9 ± 4.6	− 3.3 ± 4.21	3.029	**0.004**	0.351	0.727
Word rhyming	25.8 ± 5.4	28.4 ± 5.7	− 2.6 ± 5.40	1.873	0.066	25.8 ± 5.9	26.8 ± 5.8	− 1.0 ± 5.81	0.684	0.497	1.141	0.258
Visual tracing	10.5 ± 3.2	13.6 ± 3.3	− 3.1 ± 3.24	3.815	** < 0.001**	10.5 ± 3.3	13.5 ± 3.9	− 3.0 ± 3.37	3.322	**0.002**	0.121	0.904
Wisconsin card sorting test	50.4 ± 10.4	57.4 ± 10.7	− 7.0 ± 10.41	2.654	**0.010**	50.2 ± 10.3	62.4 ± 10.0	− 12.2 ± 10.01	4.807	** < 0.001**	2.037	**0.046**

*t1* and *p1* compared the post-intervention with the pre-intervention among cases or controls

*t2* and *p2* compared the difference between cases and controls

The bold values represent statistically significant values less than 0.05

### Safety

The adverse events that were observed included behavioral changes (e.g., drowsiness, mood instability, irritability, fatigue and dizziness), neuropsychiatric symptoms (e.g., headache), gastrointestinal reactions (e.g., loss of appetite, nausea and diarrhea), and rashes, but all of these adverse events were of mild intensity. The incidence of adverse events was 18.75% in the LEV group and 21.88% in the OXC group, with no statistically significant inter-group difference (*p* = 0.200) (Table 4).

**Table 4:** Comparison of adverse events between two groups after intervention

	Levetiracetam (*n* = 32)	Oxcarbazepine (*n* = 32)	*p*
General symptoms	1 (3.13)	1 (3.13)	0.472
CNS	1 (3.13)	2 (6.25)	1.000
Behavior	4 (12.50)	6 (18.75)	0.491
Airways	0 (0.00)	1 (3.13)	1.000
Gastrointestinal	2 (6.25)	2 (6.25)	1.000
Bones and muscles	0 (0.00)	1 (3.13)	1.000
Others	2 (6.25)	2 (6.25)	1.000
Total*	6 (18.75)	7 (21.88)	0.756

*If one patient had two or more complaints, they only counted once in calculation of total incidence rate of adverse events

## Discussion

In this study, we investigated the efficacy of LEV and OXC monotherapy in the treatment of children with BECTS, and the effects of these treatments on their intellectual and cognitive development. The results showed that both LEV and OXC effectively treated BECTS by reducing the frequency and degree of onset of seizures, without obvious adverse events and had little effect on the development of children’s intelligence and cognitive ability. However, our study also found that OXC was more efficacious and had better effects on children’s intellectual and cognitive development.

Currently, most advocates of the use of drugs to treat epilepsy favor monotherapy, because a single AED is easier for patients to take, and because multiple AEDs may interact and give rise to more adverse events [15]. For example, a multicenter clinical trial has shown children with BECTS treated with multi-AED combination therapy may exhibit mild abnormalities in imaging and bilateral EEG discharge [16]. In addition, a review of the adverse events of six AEDs commonly used in southern China from 2003 to 2015 revealed that the incidence of severe adverse events caused by treatment with one, two or three or more AEDs was 9.9, 10.0 or 19.6%, respectively. This suggests that multiple AED therapy may significantly increase the incidence of severe adverse events, and that this increase is largest when three or more AEDs are used in combination [17]. Thus, for the treatment of children with BECTS, it is crucial to select an appropriate and effective AED that has the least adverse effects on cognition, considering the rapid development of intelligence and cognitive ability that occurs during childhood.

In this study, the efficacy of LEV and OXC as monotherapy for children with BECTS was examined for 6 months, with these two AEDs selected due to their having little adverse effect on children’s cognitive development. The efficacy and effects of LEV and OXC on children’s cognitive function were compared over this period to determine which AED had the least adverse effect on children’s cognitive function. The minimal effects of these AEDs on the cognitive function of children may be due to the fact that LEV has neural center selectivity, while OXC selectively inhibits voltage-dependent ion channels, which preserves the function of postsynaptic glutamate transmitters [18].

We comprehensively evaluated and compared the intelligence and cognitive function development of the two treatment groups on the basis of eight indicators. The results showed that the cognitive abilities of both groups were improved compared with children before treatment, which proved that LEV and OXC helped to improve (rather than adversely affected) the cognitive development of children. Across all indicators, the OXC group was superior to the LEV group, in terms of treatment effectiveness, processing speed, spatial skills and executive function, and these differences were significant (*p* < 0.05) [19]. In a previous study of initial anticonvulsant monotherapy for the routine care of children and adolescents, it was also found that LEV failed more frequently than OXC, due to a lack of efficacy, and a significantly greater increase in the domain score of agitation/aggression and irritability was observed in the LEV group compared to the OXC group [20]. However, other studies have found that LEV monotherapy was comparable with OXC monotherapy for the treatment of adults with newly diagnosed focal epilepsy, in terms of long-term effectiveness, safety and tolerability [12, 21]. The difference might be due to different effects of LEV and OXC on various populations and types of epilepsy.

We also observed a decrease in the epileptic discharge detected by EEG in the two groups after treatment. During the follow-up period, the EEGs of the treated children at 3 and 6 months were improved, and the improvement efficiency of the LEV group was higher than that of the OXC group. However, this inter-group difference was not significant (*P* > 0.05), which was consistent with previous reports [22]. In contrast, recent studies have found that OXC treatment may fail to improve or may even worsen EEG results [23]. Some studies have suggested that OXC has a greater effect on EEG background activity, whereas other have suggested that the efficacy of the two drugs is similar [24].

The advent of second-generation AEDs has increased the range of drugs available for epilepsy treatment. These AEDs have received increasing attention and acceptance due to their improved pharmacokinetic properties and fewer drug interactions. LEV and OXC are two representative second-generation AEDs that are used to treat BECTS. In an open, multicenter, randomized phase IV trial in Korea, LEV monotherapy and OXC monotherapy were compared for the treatment of newly diagnosed adult focal epilepsy, in terms of long-term efficacy (over 50 weeks), safety and tolerability. The results showed that LEV monotherapy led to a lower failure rate and a lower frequency of seizures than OXC monotherapy, which demonstrated that LEV was a useful initial monotherapy option for newly diagnosed patients with local epilepsy [12]. However, the results of the study described herein show that the effective treatment rate of the OXC group was 78.12% and that of the LEV group was 53.12%, and that the frequency of monthly seizures in the OXC group was much lower than that in the LEV group at 6-month follow-up. Crucially, this clinical difference was statistically significant (*p* = 0.035), confirming that the treatment efficacy of OXC was greater than that of LEV, which was consistent with the efficacy reported in previous studies [25, 26].

This study has certain advantages. Although there have been previous studies on LEV and OXC, these were not comprehensive, and did not closely examine the effects of these AEDs on the cognitive ability of children, such as those undergoing treatment for BECTS. Therefore, in this study, LEV or OXC monotherapy was used to treat children with BECTS, and a detailed comparative analysis was made of the ability of each of these AEDs to clinically control epileptic seizures, and their ability to improve children’s cognitive ability. We used eight indicators to compare the overall cognitive development ability of the two treatment groups, and the results showed that the cognitive abilities of the two groups were improved compared with those before treatment, and that the cognitive abilities of the OXC group were better than those of the LEV group. In addition, we also compared the EEG improvement generated by each of these AEDs, and their adverse effects in children. This enabled us to objectively evaluate the advantages and disadvantages of LEV and OXC, to provide guidance for the clinical treatment of children with BECTS. This is the first comprehensive study of the effects of LEV and OXC on the intellectual and cognitive function of children with BECTS, and also examined the efficacy and safety of these two drugs for the treatment of BECTS in children. Such a breadth of data is rarely reported, even in countries with broader research environments.

However, this study has some deficiencies. Due to manpower and time limitations, the follow-up time and sample size were relatively insufficient, and thus, the long-term efficacy of LEV and OXC in children with BECTS requires further study. In addition, we did not include a control group of healthy children.

More new AEDs must be developed to treat children with epilepsy. In addition, clinical evidence must be continually updated, and treatment-related guidelines must be formulated and revised to achieve effective control of seizures in children without affecting their cognitive development or quality of life.

In summary, LEV and OXC, two second-generation AEDs that have little adverse effect on cognitive ability, were shown to effectively treat epilepsy and improve cognitive ability in children. OXC treatment was found to be more effective than LEV treatment, especially in improving children’s cognitive function, which shows that OXC is worthy of clinical application in this context.
